# New prediction categories in CASP15

**DOI:** 10.1002/prot.26515

**Published:** 2023-06-12

**Authors:** Andriy Kryshtafovych, Maciej Antczak, Marta Szachniuk, Tomasz Zok, Rachael C. Kretsch, Ramya Rangan, Phillip Pham, Rhiju Das, Xavier Robin, Gabriel Studer, Janani Durairaj, Jerome Eberhardt, Aaron Sweeney, Maya Topf, Torsten Schwede, Krzysztof Fidelis, John Moult

**Affiliations:** ^1^ Genome Center University of California Davis California USA; ^2^ Institute of Computing Science, Poznan University of Technology Poznan Poland; ^3^ Institute of Bioorganic Chemistry, Polish Academy of Sciences Poznan Poland; ^4^ Biophysics Program, Stanford University School of Medicine Stanford California USA; ^5^ Biochemistry Department Stanford University School of Medicine Stanford California USA; ^6^ Howard Hughes Medical Institute, Stanford University Stanford California USA; ^7^ Biozentrum, University of Basel Basel Switzerland; ^8^ SIB Swiss Institute of Bioinformatics Basel Switzerland; ^9^ Centre for Structural Systems Biology (CSSB), Leibniz‐Institut für Virologie (LIV) Hamburg Germany; ^10^ Universitätsklinikum Hamburg Eppendorf (UKE) Hamburg Germany; ^11^ Institute for Bioscience and Biotechnology Research, Department of Cell Biology and Molecular genetics, University of Maryland Rockville Maryland USA

**Keywords:** 3D structure prediction, CASP15, protein structure, protein–ligand complexes, RNA structure

## Abstract

Prediction categories in the Critical Assessment of Structure Prediction (CASP) experiments change with the need to address specific problems in structure modeling. In CASP15, four new prediction categories were introduced: RNA structure, ligand‐protein complexes, accuracy of oligomeric structures and their interfaces, and ensembles of alternative conformations. This paper lists technical specifications for these categories and describes their integration in the CASP data management system.

## 1 INTRODUCTION

Critical Assessment of Structure Prediction (CASP) is a well‐established experiment for tracking progress in computational methods for calculating protein structure from amino acid sequence and related challenges.^1^, ^2^, ^3^, ^4^ While the core of CASP has remained unchanged since its inception, the scope changes frequently, with some categories becoming obsolete while new ones are introduced. Following the major advances in single protein structure modeling seen at CASP14, three categories were discontinued for round 15: contact prediction, refinement, and third‐party model accuracy estimation for monomeric targets. At the same time, discussions among the organizers and advisory board members as well as a survey of the modeling community established interest in the new categories of RNA structure, protein–ligand complexes, accuracy of oligomeric structures and their interfaces, and ensembles of alternative conformations. CASP launched pilot experiments in all these areas, exploring, where possible, collaborations with groups involved in the existing or recently discontinued related experiments. In CASP15, the new categories have yielded interesting results, and their assessment is discussed in dedicated papers in this journal special issue.^5^, ^6^, ^7^, ^8^ The main goal of this paper is to address technical issues of introducing the new categories in CASP and serve as a point of reference for format specifications and details on how the experiment was conducted.

## 2 METHODS

Introducing a new category into CASP requires planning data workflows, designing formats and technical parameters for new types of models, and incorporating those into the existing CASP infrastructure. Sections 2.1–2.4 describe the implementation details for four new CASP15 categories.

### 2.1 RNA structure prediction

Prediction of RNA structure from nucleic sequence is a challenging task as RNA molecules, like proteins, can fold into a wide variety of 3D shapes. Several research groups have been actively working in this area, and in 2010 Eric Westhof pioneered a CASP‐like RNA‐Puzzles challenge^9^ to track the state of the art in the RNA structure prediction and provide a forum for discussing methodological advances. Over the course of 12 years (2010–2021) there were 22 evaluated RNA‐Puzzles challenges, which attracted the attention of around 10 returning participants.^10^ In 2022, on the initiative of Rhiju Das, Eric Westhof and CASP organizers, the RNA‐Puzzles joined forces with CASP, and RNA structure prediction became a prediction category in CASP15. This helped expand the target and predictors base of the RNA‐modeling experiment (12 targets, 25 research groups in CASP15), stimulate development of new RNA prediction methods through the exchange of ideas and techniques with the protein prediction community, where deep learning methods recently made a significant impact on the modeling accuracy,^11^, ^12^ increase visibility of the field, and use CASP's standardized platform for managing predictions and evaluating and comparing different prediction methods.

To incorporate RNA prediction into CASP, we adhered as closely as possible to the requirements and recommendations of the RNA‐Puzzles experiments.^9^

#### 2.1.1 RNA prediction format (https://predictioncenter.org/casp15/index.cgi?page=format#TS)

Similarly to protein structure prediction, a CASP RNA submission file starts with the CASP header including format specification code, target identifier, author identifier, and description of methods used for modeling. The file can include up to five RNA 3D models, each encompassed by the MODEL/END keywords. Models are formatted according to the established standards of the RNA‐Puzzles community:^9^

- 3D coordinates are provided for the complete list of atoms for all nucleotides from the target FASTA file;
- only natural nucleotides (A, C, G, U) are allowed;
- if present, modified monomeric units are transformed into unmodified ones by discarding atypical atoms;
- only atoms from the following sets—(C2, C4, C6, C8, N1, N2, N3, N4, N6, N7, N9, O2, O4, O6) for nucleobases, and (C1′, C2′, C3′, C4′, C5′, O2′, O3′, O4′, O5′, OP1, OP2, P) for sugar‐phosphate backbone are allowed.

In case of protein‐RNA complexes, protein chains are designated with letters (A, B, C, …) and RNA chains with numbers (0, 1, 2, …).

An example of RNA prediction is provided in Example 3 on the CASP15 format page https://predictioncenter.org/casp15/index.cgi?page=format.

#### 2.1.2 Preparation of targets and model templates

The CASP organizers prepare a FASTA file with the sequence of targeted RNA. The file begins with a header containing target ID (e.g., >R1117) and chain IDs (i.e., numbers from 0 to 9) of all strands in the target structure. The body of the file includes nucleic acid sequence(s). In addition, the organizers generate a 3D structure template using the RNA‐Puzzles formatting tool.^13^ The template is a PDB file containing all the required ATOM records with zeroed coordinate values. The information on targets is communicated to participating groups via the CASP web portal (e.g., https://predictioncenter.org/casp15/target.cgi?id=30&view=all).

Prior to submission, predictors can verify compatibility of their models with the provided templates by running the RNA‐Puzzles tool that checks the number and ordering of residues and atoms in the submission.^13^ If a prediction file does not comply with the requirements, error messages are reported to a log file. Non‐compliant files can be reformatted with the rna_pdb_toolsx.py script available from the rna‐tools toolbox.^13^, ^14^

#### 2.1.3 Setting the acceptance system

At the target release time, each target is assigned a prediction time window, which is typically 3 days for servers and 3 weeks for expert groups. RNA structure models are accepted within the specified prediction window via email or dedicated CASP prediction submission webform. The CASP submission system automatically checks submissions for compliance with the deadlines and format requirements and provides feedback to predictors. The prediction format is checked with the same tools used to generate model templates (Section 2.1.2). If a prediction is rejected, an error message is sent to the submitter, and they have until the target deadline to fix the reported issue(s) and resubmit. Accepted predictions are stored in the CASP system and eventually evaluated after the target structure becomes available.

The same submission rules apply to other prediction categories discussed further in this paper.

#### 2.1.4 RNA evaluation measures

**FIGURE 1:**
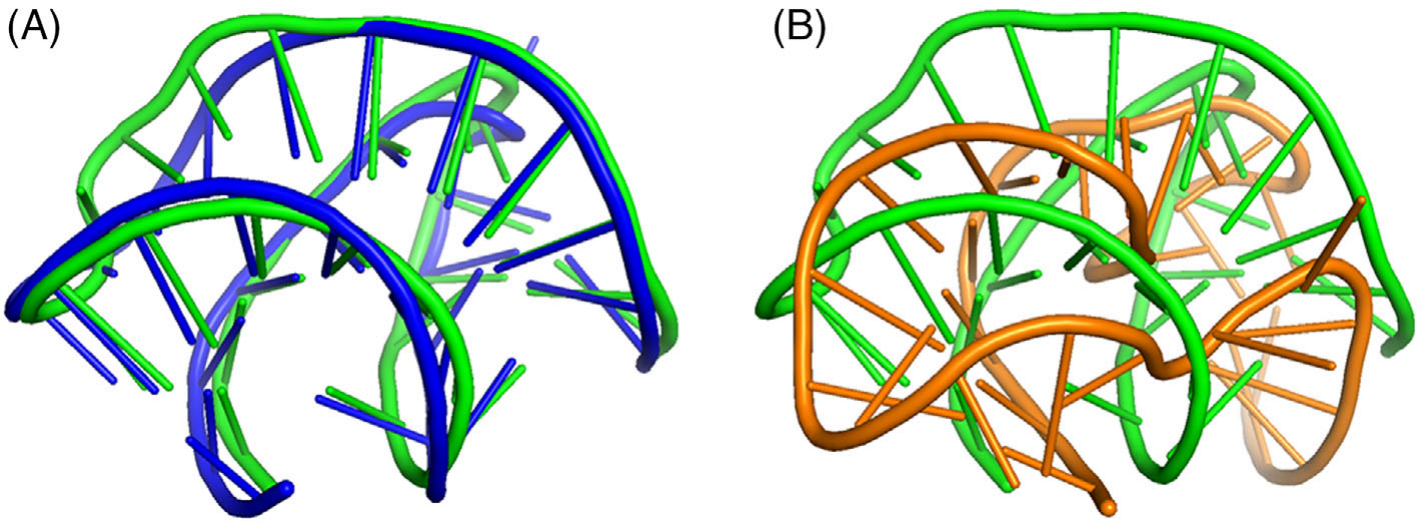
Two models of PreQ1 class I type III riboswitch superimposed with the target structure (R1117, green). Model R1117TS119_3 (orange, panel B) correctly reproduces all canonical base pairs at the same time not reproducing any non‐canonical interactions and having the RMSD of 9.90 Å. Model R1117TS287_2 (blue, panel A) misses 7% of canonical base pairs but is a much better model with the RMSD of 2.25 Å and non‐canonical interactions reproduction rate of 39%. RMSD scores were computed on the 2–30 fragment.

Predictions in the RNA category are assessed by checking their geometric plausibility and comparing them with target structures. When alternative target structures were available, we reported the best score per model. Evaluation measures include Clashscore,^15^ Root Mean Square Deviation (RMSD),^16^ Local Distance Difference Test (lDDT),^17^ Template Modeling score (TM‐score),^18^ and Global Distance Test‐Total Score (GDT‐TS).^19^ These are commonly used measures in protein‐CASP evaluation that are also adopted here for RNA evaluation. However, none of these measures are suitable for assessing RNA‐specific components, like canonical (G‐C, A‐U, G‐U), non‐canonical, and stacking interactions between the nucleobases that contribute to RNA folding and stabilization. Proper prediction of only canonical interactions is usually insufficient to obtain a good model of an RNA molecule (example in Figure 1), while prediction of non‐canonical interactions is very valuable but hard to achieve due to high computational demands. We additionally consider an RNA‐specific measure, Interaction Network Fidelity (INF),^13^, ^20^ which evaluates different types of RNA interactions in models. Calculation of these measures requires prior determination of RNA interactions from the atomic coordinates. This is done using 2D structure annotators such as RNAView,^21^ MC‐Annotate,^22^ ClaRNA^23^ or FR3D,^24^ which provide base pairs and their classification.^25^ Given two sets of interactions, one for the model and another for the target, we identify true positives (correctly predicted base pairs), false positives (unpredicted base pairs), and false negatives (incorrectly predicted base pairs), and then calculate the INF score as the Matthews correlation coefficient.^26^ The score ranges from [0.0, 1.0], with higher scores indicating better prediction of base–base interactions. The INF score is determined for all interactions (INF_all), and separately for canonical (Watson‐Crick, INF_WC), non‐canonical (non‐Watson‐Crick, INF_nWC), and stacking (INF_stacking) interactions.

Many RNA targets were derived from low‐to‐medium resolution (4–7 Å) cryo‐EM maps. To remove bias and modeling uncertainties potentially present in the reference structures, we additionally evaluated predictions by directly comparing them to maps. After docking models into maps, predictions were scored with five different measures: CC_mask_ (cross‐correlation masked by the area around the model), CC_peaks_ (cross‐correlation masked by the model and the highest density peaks in the map), MI (mutual information), SMOC (segment‐based Mander's overlap coefficient), and AI (atomic inclusion).^27^, ^28^ It is worth noting that model‐independent measures are known to overinflate scores for compressed models with unrealistic geometries. Thus, when using these scores, it is important to first filter out models with poor topology scores or, alternatively, combine these scores with geometric scores, such as Clashscore.

### 2.2 Protein–ligand complexes

The ligand topic is not new to CASP: in CASPs 6 through 10 predicting ligand binding sites was a sub‐challenge in the function prediction category.^29^, ^30^, ^31^, ^32^ Given the recent advances in the accuracy of protein modeling methods,^11^, ^12^ CASP organizers decided to include prediction of protein‐ and RNA‐small molecule ligand complexes into the scope of CASP15 experiment hoping to boost development of methods in this area. Participants are provided with the sequence and stoichiometry of protein (or RNA) receptors, Simplified Molecular Input Line Entry System (SMILES) codes of bound ligands, and are asked to predict structures of protein– (RNA‐)ligand complexes.

#### 2.2.1 Macromolecule–ligand complex prediction format (https://predictioncenter.org/casp15/index.cgi?page=format#LG)

One important requirement for the ligand prediction format was the need to encode atom connectivity in a robust and reliable manner, as the correct atom connectivity is required for symmetry correction, a necessary step in accurate ligand assessment. Unfortunately, the PDB format, which is commonly used in CASP, is not able to reliably encode connectivity for arbitrary ligands. The MDL molfile format^33^ is a common format for ligands which was used in earlier ligand docking challenges such as D3R.^34^, ^35^, ^36^, ^37^ This is a text‐based, fixed column format that besides atom coordinates also encodes the bonds. Unlike the PDB format, atoms are not named and only identified by their element and connectivity. The format allows reporting additional properties such as charge, valence, or isotope, but those were not required nor used here. Bonds between atoms are encoded explicitly, one by line, together with the bond type (single, double, triple, or aromatic) and stereochemistry. The format also includes header lines, a COUNTS line, which can help check the integrity of the file, and an M END line which indicates the end of the ligand data.

For CASP15, we devised a hybrid submission format where the receptor's model (protein or RNA) and ligand model are submitted as separate files in the same spatial frame of reference. The receptor is submitted in the PDB format, while the ligand in MDL (see below for details). Similarly to the regular protein structure submission, a CASP ligand submission (LG format) starts with a CASP header including format specification code, target identifier, author identifier, and description of the modeling method. Two new keywords are introduced: the LIGAND keyword, which defines ligand name and the beginning of the ligand data, and the POSE keyword, which defines the pose number for the selected ligand. Participants are allowed to submit up to five poses of a given ligand for a selected receptor model.

An example of LG prediction is provided in Example 6 on the CASP15 format page.

#### 2.2.2 Preparation of targets and model templates

A FASTA file of the receptor sequence is prepared by the CASP organizers. For known small molecules, SMILES are retrieved from the PDB component dictionary. In the case of novel small molecules (not present in the PDB component dictionary), SMILES are provided by the experimentalists. In both cases, SMILES are compared and modified based on those derived from the PDB coordinates. If necessary, stereochemistry is assigned using the *AssignStereochemistryFrom3D* function from RDKit, and the protonation state adjusted by manually editing the SMILES based on the visual inspection of protein‐ligand interactions.

The relevance of each small molecule is decided case by case. Only biologically relevant small molecules are retained. Common crystallographic reagents and ions are ignored if not interacting with the small molecules or part of a structural motif (e.g., zinc binding motif).

A script to prepare prediction templates (MDL files) is provided by the CASP organizers. It is implemented in python 3 and RDKit python bindings (http://www.rdkit.org/). The script initially converts the input SMILES strings to RDKit Mol objects using the *rdkit.Chem.MolFromSmiles* method. At this stage, the Mol objects contain only the information related to small molecule properties, like atom types and bond formation. Coordinate section is added to the Mol objects using the RDKit's ETKDG method.^38^ Subsequently, the Mol objects are written to the MDL‐formatted file,^33^ which can be used as a ligand submission template.

#### 2.2.3 Setting up the acceptance system

Validation of ligand predictions is performed with scripts written in python 2.7 and RDKit. Initial checks verify the CASP header section (availability and correctness of PFRMAT, TARGET, AUTHOR, and MODEL/END records). Once submissions have passed this phase, ligand models are converted to RDKit Mol objects and compared with the template for downstream evaluation. Each molecule in the submitted file is validated by comparison with a reference Mol object generated from the corresponding SMILES string as described above. To validate the submissions, comparisons of the following parameters are undertaken:

- number of atoms and their types,
- number of bonds,
- bond types and atom types in bond pairs (e.g., C—C Single, or C=O Double).

Additionally, to account for atom connectivity and chirality in submitted models, the maximum common substructures between the submitted and reference ligands are calculated using the *FindMCS* function in RDKit. To pass the validation, a molecule must have a maximum common substructure equal to the number of atoms in the reference model.

Finally, a validation report is created showing the results of the validation process to aid in troubleshooting invalid submissions.

#### 2.2.4 Macromolecule–ligand complex evaluation measures

Previous ligand docking challenges like Teach Discover Treat (TDT),^39^ Continuous Evaluation of Ligand Prediction Performance (CELPP)^40^ and Drug Discovery Data Resource (D3R)^34^, ^35^, ^36^, ^37^ have used two main types of metrics to assess how well participants can model receptor–ligand complexes. These evaluated how close a predicted ligand is to the target within the binding site in absolute terms with the RMSD metric, and how well the native receptor–ligand interactions are reproduced. CASP experiment brings additional assessment challenges: (1) because the receptor structure is not given but rather modeled, ligands in the model and reference complexes can be bound to different configurations of binding sites, and thus calculation of any superposition‐based scores requires preliminary alignment of binding pockets with ligands in two complexes, which is not a trivial task; (2) chain mapping needs to be established; (3) incomplete ligands in some targets require partial graph matching for the symmetry correction; and (4) multiple copies of ligands in the targets and models have to be mapped (assigned) uniquely, in order to avoid scoring target or predicted ligands multiple times.

To address these challenges, we developed two scores, which are described in more detail in the CASP15 Ligand Assessment paper.^7^ The Binding‐Site Superposed, Symmetry‐Corrected Pose Root Mean Square Deviation (BiSyRMSD) score defines the binding sites and the superpositions to compute RMSDs between target's and model's ligands. The Local Distance Difference Test for Protein–Ligand Interactions (lDDT‐PLI) measure assesses how well native contacts between the receptor and the ligand are reproduced in the model with an lDDT‐based metric and symmetry correction. When used in combination, these scores give a better account of modeling receptor–ligand complexes.

### 2.3 Estimation of model accuracy for oligomeric targets

The estimation of model accuracy (EMA) category has been an integral part of every CASP experiment starting with CASP7.^41^, ^42^, ^43^, ^44^, ^45^, ^46^, ^47^, ^48^ It has attracted the attention of many developers, with over 70 methods tested in the previous CASP experiment.^48^ An emphasis on the importance of this category led to very positive developments in protein structure prediction as modelers now routinely integrate quality estimates into their modeling pipelines. In particular, the CASP14‐winning AlphaFold2 method offers reliable estimates of global and local accuracy of their models.^10^, ^11^

In CASP15, the focus of the EMA category shifted from predicting accuracy of single‐sequence proteins to multi‐molecular complexes.

#### 2.3.1 Model accuracy prediction format (https://predictioncenter.org/casp15/index.cgi?page=format#QA)

For global (whole model) accuracy prediction (QMODE1), participants are asked to submit a fold similarity score (SCORE, in 0–1 range), which estimates the similarity of model's overall fold to the target's one, and an interface similarity score (QSCORE, also in 0‐1 range), which evaluates reliability of quaternary structure interfaces. Submitting the QSCORE is optional, and predictors can skip it by putting ‘X’ symbol in the corresponding place of a QA prediction (see the link above). In QMODE2 (local accuracy), in addition to the QMODE1 scores, the predictors are asked to assign confidence scores to the interface residues of the model, indicating their likelihood of being present in the native structure's interface. Interface residues are identified as having contact with at least one residue from a different chain, with a Cβ–Cβ distance not exceeding 8 Å (or Cα, if the residue is glycine).

Examples of EMA predictions in QMODE1 and QMODE2 are provided in Example 5 on the CASP15 format page.

#### 2.3.2 Submission collecting process

EMA predictions in CASP15 are requested for all (and only) multimeric targets. In contrast with previous CASPs, EMA targets are released after all models (and not only server models) are collected on the corresponding structure prediction target. A tarball with assembly predictions from all CASP groups is created the next day after the TS target closure, and a link to the tarball file is pushed to the EMA servers and posted at the CASP15 website. All EMA groups, regardless of their type (i.e., ‘server’ or ‘human’) have 2 days to return accuracy estimates for TS models included in the tarball file. The predictions are checked with the verification scripts, and successful predictions are saved for subsequent evaluation.

#### 2.3.3 EMA evaluation measures

Global predictions were compared with established evaluation metrics possessing the desired attributes. This is the oligomeric Template Modeling score (TM‐score)^49^ for overall topology (SCORE) and the contact based QS‐score^50^ which is interface centric (QSCORE). To ensure a comprehensive evaluation, these metrics were supplemented with additional measures. An oligomeric GDT‐like score, referred to as oligo‐GDTTS, was employed for overall topology analysis, and a variant of the interface centric DockQ score.^51^ Notably, DockQ evaluates pairwise interfaces, necessitating the introduction of a weighted average metric—termed DockQ‐wave—to effectively score higher‐order complexes. Local predictions were compared against the per‐residue lDDT^17^ and CAD (AA‐variant)^52^ scores, which assess the accuracy of relative atom positions in the neighborhood, including neighboring chains. Conceptually the scores are contact‐based, but do not penalize for added contacts, which is relevant in case of incorrect interfaces. To address this limitation, two novel local variants of the QS‐score and DockQ have been introduced: PatchQS and PatchDockQ. All evaluation metrics are described in detail in the CASP15 EMA Assessment paper.^6^

### 2.4 Ensembles of alternative conformations

Following the success of deep‐learning methods for single structures, it is increasingly important to assess methods for predicting ensembles of alternative conformations. While deep learning and other methods have the potential to generate ensembles in some circumstances, these abilities have never been rigorously tested. In CASP15, we made a first attempt to include this category. For CASP purposes, we categorize ensembles^8^ as: (1) Cases where a macromolecule populates multiple conformations under the same environmental and chemical conditions (including intrinsically disordered proteins or parts of proteins; vibrational motion; local alternative conformations; ‘ghost’ conformations which are present at low level but are dominant in other conditions; and folding intermediates.). (2) Cases where a macromolecule adopts different conformations in response to environment or chemical change (ligand binding; macromolecular complex formation; post‐translational modification; mutations; and crystal, pH and other environmental changes). A third category of ensembles we consider is the set of conformations consistent with the experimental data. The latter is an increasingly important category both because of the now common high accuracy of the computed structures and the inclusion of lower resolution data in CASP.

Targets for alternative conformers do not require separate prediction formats as they are 3D structures routinely processed in CASP, but they do require a mechanism for submitting multiple models. In CASP15, this need was handled in two different ways. In some cases, different alternative conformations were treated as separate targets. In particular,

- two targets were assigned for modeling an isocyanide hydratase represented by a wild‐type structure (target T1110) and its one‐point mutant T1109, where amino acid D183 was changed to A183,
- two targets (R1107 and R1108) were assigned for modeling human and chimpanzee CPEB3 ribozymes, which differ by a single nucleic acid A30 (human) → G30 (chimpanzee),
- two pairs of targets (TR1189 and TR1190) were assigned for modeling complexes of the metabolite repressor protein (RsmA) and a non‐coding RNA (RsmZ). Both complexes contain one RNA molecule but different number of protein molecules (six in TR1189 and four in TR1190),
- five targets (T1158v0‐v4) were assigned for modeling a type IV ABC transporter, where five different conformations have been observed, depending on environmental conditions (ligand binding).

In other cases, participants were encouraged to submit multiple conformers using the standard CASP five models target format. This approach was used for

- three kinases (CASP targets T1195–T1197), each of which has two to three sets of experimental coordinates representing different conformations,
- the Holliday Junction complexes (targets T1170, H1171, H1172), some subunits of which are deformed due to the contact with DNA and other protein molecules in the complex,
- RNA origami target R1138, which was solved in a kinetically trapped young state and the mature state,
- SL5 domain of the RNA betacoronavirus structure BtCoV‐HKU (CASP target R1156), where one of the helices accepted multiple relative conformations with respect to the remainder of the structure.

## 3 CONCLUSION

This paper summarizes the technical aspects of new prediction categories in CASP15 and can serve as a reference point for future predictors. The systems for handling RNA/ligand/multimer‐EMA predictions in CASP15 were implemented shortly before the start of the experiment and, as such, were not extensively tested. Based on the feedback from CASP15 assessors and predictors, we plan to improve the system by further automating the process of verifying RNA and ligand submissions and ensuring better compliance of models with the required format. This investment of time and effort should pay off at the assessment stage with a more seamless evaluation of predictions. Since the PDB format is becoming obsolete, we will motivate predictors and developers of the evaluation measures used in CASP to support the PDBx/mmCIF format in their tools and methods. For the RNA category, we will work to further incorporate and standardize evaluation measures which assess the base–base interaction network. This will require setting community‐accepted standards in identification and annotation of RNA interactions.^22^ Further, we will work to improve measures comparing predictions to raw data to assess low‐resolution targets. For the ligand prediction category, we need to improve the set of targets with more interesting ligand modeling challenges, possibly by tapping into unreleased structures from pharmaceutical companies. We will also improve the acceptance system by validating the identity of submitted ligands in a more robust manner, and we would like to consider introducing metrics for self‐assessment of the accuracy of ligand poses within the receptor. For evaluating ensembles of alternative conformations, we will explore acceptance of multi‐model entities, with optional probability weights, as submissions.

## AUTHOR CONTRIBUTIONS

**Andriy Kryshtafovych:** Conceptualization; methodology; software; validation; project administration; writing – review and editing; writing – original draft; supervision; data curation. **Maciej Antczak:** Methodology; software; writing – original draft. **Marta Szachniuk:** Methodology; software; writing – original draft. **Rachael C. Kretsch:** Methodology; software; writing – original draft. **Ramya Rangan:** Software; writing – original draft. **Phillip Pham:** Software; writing – original draft. **Rhiju Das:** Software; methodology; conceptualization; writing – original draft; supervision. **Xavier Robin:** Methodology; software; writing – original draft. **Gabriel Studer:** Methodology; software; writing – original draft. **Janani Durairaj:** Software; writing – original draft. **Jerome Eberhardt:** Methodology; software; data curation. **Aaron Sweeney:** Software; methodology; data curation; writing – original draft. **Maya Topf:** Supervision; methodology; writing – original draft; funding acquisition; project administration. **Torsten Schwede:** Writing – original draft; supervision; funding acquisition; project administration. **Krzysztof Fidelis:** Supervision; project administration; resources; funding acquisition; writing – original draft. **John Moult:** Conceptualization; methodology; supervision; project administration; writing – original draft; writing – review and editing.

### PEER REVIEW

The peer review history for this article is available at https://www.webofscience.com/api/gateway/wos/peer-review/10.1002/prot.26515.
